# Perspectives from leadership and frontline staff on telehealth transitions in the Los Angeles safety net during the COVID-19 pandemic and beyond

**DOI:** 10.3389/fdgth.2022.944860

**Published:** 2022-08-09

**Authors:** Alejandra Casillas, Cristina Valdovinos, Elizabeth Wang, Anshu Abhat, Carmen Mendez, Griselda Gutierrez, Jennifer Portz, Arleen Brown, Courtney R. Lyles

**Affiliations:** ^1^ Division of General Internal Medicine and Health Services Research, UCLA David Geffen School of Medicine, Los Angeles, California, United States; ^2^ UCLA David Geffen School of Medicine, Los Angeles, California, United States; ^3^ Harbor-UCLA Medical Center, Los Angeles County Department of Health Services, Los Angeles, California, United States; ^4^ University of Colorado School of Medicine, Denver, Colorado, United States; ^5^ UCSF Departments of Medicine and Epidemiology and Statistics, San Francisco, California, United States; ^6^ UCSF Center for Vulnerable Populations, San Francisco General Hospital, San Francisco, California, United States

**Keywords:** telehealth, telemedicine, digital divide, digital health disparities, safety net, vulnerable populations, COVID-19, community-partnered participatory research, qualitative research

## Abstract

**Objectives:**

The start of the COVID-19 pandemic led the Los Angeles safety net health system to dramatically reduce in-person visits and transition abruptly to telehealth/telemedicine services to deliver clinical care (remote telephone and video visits). However, safety net patients and the settings that serve them face a “digital divide” that could impact effective implementation of such digital care. The study objective was to examine attitudes and perspectives of leadership and frontline staff regarding telehealth integration in the Los Angeles safety net, with a focus on telemedicine video visits.

**Methods:**

This qualitative study took place in the Los Angeles County Department of Health Services (LAC DHS), the second-largest safety net health system in the US. This system disproportionately serves the uninsured, Medicaid, racial/ethnic minority, low-income, and Limited English Proficient (LEP) patient populations of Los Angeles County. Staff and leadership personnel from each of the five major LAC DHS hospital center clinics, and community-based clinics from the LAC DHS Ambulatory Care Network (ACN) were individually interviewed (video or phone calls), and discussions were recorded. Interview guides were based on the Consolidated Framework for Implementation Research (CFIR), and included questions about the video visit technology platform and its usability, staff resources, clinic needs, and facilitators and barriers to general telehealth implementation and use. Interviews were analyzed for summary of major themes.

**Results:**

Twenty semi-structured interviews were conducted in August to October 2020. Participants included LAC DHS physicians, nurses, medical assistants, and physical therapists with clinical and/or administrative roles. Narrative themes surrounding telehealth implementation, with video visits as the case study, were identified and then categorized at the patient, clinic (including provider), and health system levels.

**Conclusions:**

Patient, clinic, and health system level factors must be considered when disseminating telehealth services across the safety net. Participant discussions illustrated how multilevel facilitators and barriers influenced the feasibility of video visits and other telehealth encounters. Future research should explore proposed solutions from frontline stakeholders as testable interventions towards advancing equity in telehealth implementation: from patient training and support, to standardized workflows that leverage the expertise of multidisciplinary teams.

## Introduction

Digital health care has expanded over the past decade, largely driven by the financial incentives of the Health Information Technology for Economic and Clinical Health (HITECH) Meaningful Use program, part of federal health care reform. In the last few years, *safety net systems* (1), health systems that provide a significant level of care to minority, low-income, Limited English Proficient (LEP), and other patients from under-resourced backgrounds, have implemented digital tools for the first time. Since safety net patients already face social barriers related to the effort and cost of accessing in-person care such as taking unpaid time off from needed work, and transportation costs—digital health is an important tool for improving health equity in this group (2–9). Telehealth services like patient portals and telemedicine phone and video visits have the potential to augment access to health care and improve outcomes (10–16).

\Coupled to this, the Coronavirus-19 disease (COVID-19) pandemic propelled telehealth into the forefront as a clinical mechanism towards maintaining access to health care during a global shut down (as has occurred during other times of crisis) (17–29). However, this forced and uncharted transition immediately raised concerns about equitable access for safety net patients (3, 30, 31). Health leaders and researchers have documented how the abrupt shift from in-person visits to telemedicine with COVID-19 left many safety net health systems ill-prepared to support digital uptake (3, 31–33), especially among historically and contemporarily underserved patient groups (3, 31, 33, 34). Among those most left behind in the digital health waves are the 22.3 million LEP residents of the US (35), who already experience health access barriers due to language and literacy (36–40) that place them at higher risk for inadequate disease control, higher utilization of acute care, and poor health outcomes (41–43). A study examining Kaiser Permanente patients from 2016 to 2018 found that populations with non-English language preference were significantly less likely to access a telehealth visit than English-speakers, and that patients living in low SES areas were less likely to have a video visit than those living in higher SES neighborhoods (44). Early evidence during the COVID-19 pandemic confirmed lower rates of telehealth visits for older, non-White, and LEP patients (3, 31–33, 45).

With no established digital guidance in place for safety net settings prior to the pandemic, the evidence demonstrates that telehealth in health settings that care patients from underserved backgrounds will have to be monitored as implementation evolves—to understand and mitigate barriers to use. As such, studying telehealth implementation within safety net settings is critical for developing multi-level solutions that advance digital health equity. Therefore, to explore strategies and barriers to telehealth implementation in the safety net, we focused on the highest quality synchronous telehealth modality as the study case example—video visits—within the Los Angeles County Department of Health Services (LAC DHS), the second largest municipal healthcare system in the US. The objective of this study was to interview health system leadership and frontline clinical staff to understand their perspectives on, and experiences with the delivery of video visits during the COVID-19 pandemic, and to also inform subsequent telehealth implementation efforts in this safety net, and nationwide.

## Methods

### Study design and setting

LAC DHS forms the core of the health care safety net for indigent populations in Los Angeles County—the largest and most ethnically diverse county in the US. Over 50% of the primary care patient population is LEP (46–49). This safety net health system serves more than 10 million residents and provides over 2.5 million ambulatory visits every year across Los Angeles County. Between August and October 2020, we conducted in-depth individual interviews with LAC DHS stakeholders who, at the time, were implementing some of the first telemedicine video visits in their respective Los Angeles safety net clinics.

### Participants and recruitment

Individuals were recommended for interview by a study co-investigator (AA) who is the LAC DHS medical director for patient engagement and population health. Participants were nominated given their role in the implementation of video visit “pilots” at clinics across LAC DHS (spring 2020). Potential participants were emailed to inform them about the study. Participants could also nominate other stakeholders involved or leading the pilot video visits (e.g., snowball sampling). Those who responded and accepted were scheduled for a 1-hour video or phone interview. Each participant was offered a $50 Amazon gift card for participation. The study was approved by the UCLA and LAC DHS Institutional Review Board.

### Data collection and analysis plan

We based our interview guide on the Consolidated Framework for Implementation Research (CFIR), a conceptual framework that guides systematic assessment of multilevel contexts to identify factors that might influence implementation and effectiveness (50). The questions were also based on the published digital health literature among safety net patients in the United States (AC and CL) (32, 51–67), and further amended by our study team, which included LAC DHS stakeholders (AA, GG, CM). The interviews (conducted by AC) included questions on: the debut of video visits in clinics, the preparation and process for clinics, staff, and patients to offer, schedule, and conduct video visits, and how these video pilots evolved or changed over time, and adaptions. Questions probed around issues surrounding technology platform, usability, staff resources, clinic needs, and stakeholder perceptions of facilitators and barriers. We asked interviewees to consider these discussions for the unique strata of patients served by LAC DHS: LEP, digital/health literacy challenges, and limited access to Internet and/or Internet-connected software and devices. Interviews were digitally recorded and transcribed. Transcriptions were independently read by 3 members of the research team (AC, EW, CV) to develop a codebook. Two coders analyzed the interview transcripts (AC, CV) to organize participant quotes under unifying themes.

## Results

Of 27 stakeholders that were nominated, 20 individuals accepted and these semi-structured interviews were conducted between August–October 2020. Participants included physicians, nurses, medical assistants, and physical therapists with clinical and/or administrative roles (Table 1). Participants were either: primarily based at one of five major hospital/clinic medical centers that are part of the LAC DHS health system (DHS site) or worked within LAC DHS’ network of 26 non-hospital affiliated community health centers and community clinics all over Los Angeles (Ambulatory Care Network, ACN). Narrative themes surrounding telehealth implementation in the Los Angeles safety net are organized into patient, clinic/provider, and health system levels that correspond to constructs in the CFIR, with accompanying exemplar quotations (Table 2).

**Table 1:** Safety-net Stakeholders’ clinical role/training and operational/leadership role relevant to telehealth implementation (*n* = 20).

Clinical training	Operational role
Physician	Medical Director of Dermatology at DHS site
Physician	Chief Medical Information Officer at DHS site
Physician	Medical Director of Ambulatory Specialty Care at DHS site
Physician	Medical Director of Ambulatory Specialty Care at DHS site
Physician	Medical Director of Primary Care at DHS site
Physician	Medical Director of HIV Primary Care at DHS site
Physician	Chief Operating Officer at DHS site
Physician	Medical Director of an ACN community health center
Physician	Medical Director of an ACN community health center
Physician	Medical Director of urgent care in ACN community health center
Nurse	Clinical Nursing Director of Specialty Clinics at DHS site
Nurse	Neurology clinic nurse manager at DHS site
Nurse	Supervising Staff Nurse of Specialty clinics at DHS site
Nurse	Nurse Manager, ACN Population Health
Nurse	Clinical Nursing Director of Ambulatory Care Services at DHS site
Medical Assistant	Outpatient Care Health Education at DHS site
Medical Assistant	Outpatient Care Health Education at DHS site
Physical Therapist	Physical Therapy Clinical Supervisor at DHS site
Physical Therapist	Director of Speech Therapy and Audiology at DHS site
Physical Therapist	Director of Rehabilitation Technology at DHS site

**Table 2:** Multilevel themes addressing telehealth implementation in the safety net.

LEVEL cfir construct	Theme	Exemplar quotation by safety net stakeholder
PATIENT Outer Setting	Patient preparedness for telemedicine	*Some are very tech-savvy, some can’t get an email on their phone or don’t even have a phone. So that has been one of the challenges*. -Physician *I’m going to say 50, 60% of patients or more have challenges getting it set up. And in our communications with the nurses who have done it so far, they said sometimes it can take 30 min to an hour just to walk a patient through getting set up if they have to have them download Zoom and they have to send them a link and they have to literally walk them through the process.* –Physician *Now we have to look at the IT literacy of our patients as well. And I think that’s probably even more challenge than our health literacy for a lot of patients.* –Physician *There’s no sense in developing a really great workflow … when the patient doesn’t know the technology.* –Physician *So access is a challenge and then just knowing how to use the basic functions of the phone. Most of what we do over FaceTime or Zoom is using a front-facing camera, but if you’re working with a dermatologist or a podiatrist, you actually want to use the rear-facing camera and point the device towards your foot and so, in the middle of a Zoom call, how do you move to the other camera? Or another example that comes to mind is, okay, I’ve got you on the phone and you want me now to click on the message that I got as a text? I don’t understand how I’m supposed to app-switch from being on the phone to now going to messages and now going to Zoom and often patients will ask, “Am I supposed to hang up now and go to messages?* -Physical Therapist *But they were really basically, first, do you have a smartphone or a computer or a tablet that has a video capability that you could use for a video session? Second question is, are you functioning on data minutes or do you have Wi-Fi capability and you have unlimited data? Because of a concern that sometimes people might be paying for data per megabyte or whatever and if we’re going to have a 60-minute call, then we don’t want them to come away with a $60 phone bill as a result of it because it’s data intensive. Really, it’s do they have the technology? Are they able to download the software and let’s try out the software and make sure that you succeeded at it. And is this your phone? So if they grab their friend’s phone and do the whole and then on the day of, they don’t have a phone anymore, that’s a bit of an issue.* -Physical Therapist *Well, one of the things we really tried to impress upon the providers is you will have a successful video visit based on your patient selection. Right? Because there’s so many things involved. The patient has to be a little tech-savvy.* –Nurse *… couldn’t even figure out how to open the text messages app on his phone to see a text message. There was no chance he was going to be able to run a video visit. And he hung up on me like ten times and I was unclear whether he was purposely hanging up on me or not. And then eventually we called his daughter because we’re like, “Maybe his daughter can help him do this”* –Physician *… the barriers for our patients are the email issue and then in terms of the app, a lot of them have phones with very limited capacity. So either they don’t want to, or they can’t add an app … It’s doable, but it’s another technical barrier for them.* -Physical Therapist *… but the more I talk to patients and the more I’ve heard from our staff, with the pandemic I feel like it’s internet and Wi-Fi, or data, it almost needs to be a public utility. Our patients struggle with that. And being a pediatrician, I hear a lot of stories of students having troubles keeping up with school because they can’t connect, which is unfortunate. It shouldn’t be in this—at least in a major metropolitan city that we’re in, I think that’s one of our major connections. That’s probably what we’re going to struggle with as far as when it comes to video visits.* –Physician *Some patients, when found out they may be charged carrier fees, declined right off the bat because of that. Some patients, even though we chose them, didn’t feel comfortable doing the video visit. They were more comfortable doing a telephone visit …And ensuring the proper platforms to support the video visit, their phone, yeah.* –Nurse *Again, quality of the client’s internet can be challenging because sometimes it’s very glitchy. Their equipment sometimes the video quality—especially, for example, like for [derm] you’re looking at an atopic rash or you’re looking at some other dermatological issue that you need a close-up on and it’s pixelated and you can’t really see well—I think that is a challenge.* –Nurse *… the issue I have with it is like an equity issue that I think that some of the patients that are probably least capable of setting up video visits or some of the people most in need of that resource and it’s kind of unfair and not cool to say like, hey, here’s this great clinical resource, but we’re just going to deny it to a big swath of the patient population just because it’s too hard for us. So I think it’s on us to figure out how to make it work, not on us to figure out which patients to and to not give it to.* –Physician *It’s, how can we make this work for people? And someone said, “You know what? We should actually have IT like the Apple Genius Bar. We need a genius bar in DHS just to help people enroll in an email and get all of that.”* –Physician *Is there a packet that we should be giving to patients head of video visits or to advertise for video visits to get them prepper? Are there maybe two different packets, one for folks that are gung-ho like, “Yeah, I want to do a video visit. I know how to do that”… and then another one would be the patient who’s questioning or if they’re going to do a video visit, they really need a family member to be with them during the visit to almost accompany them to the video visit.* –Physician

* *	Patient acceptability of telemedicine	*In the beginning, patients were like, “Well, I don’t know if I feel comfortable about this.” They were unsure about having their information getting out. And we assured them that we were confidential in all of our information, just like as if they were here*. -Nurse *Maybe if it’s spreading, just if the patients are willing to do it. Some want to be seen, some want to come in, or some are afraid of this technology stuff or some just don’t have the patience to do it.”* -Medical Assistant *I think the biggest challenge for us first is just changing the culture …And I think just that lack of coming face to face and seeing the nurse and having your vital signs taken and all of that was really a culture shock for a lot of our patients that they still haven’t recovered from. And they want to come in and see us. And we keep trying to encourage I can do most of the same stuff over the phone or even over a video visit, but culturally I think that’s where we haven’t gotten to the point where we’ve really said it’s acceptable and the norm in medicine to see you over a video visit and that I can give you almost the same care … And I think there is a lot to be said just for the human connection when you’re in the same room, but the culture I think is the hardest part of sell.* –Physician *If you can’t push them into a video or a telephone visit, let them come in, it’s okay.” We will see you and then let’s slowly develop the rapport and say, “Look, okay, Mrs. Jones, I can do all of this stuff and I know I’m used to seeing you four times a year, but realistically I can see you maybe once a year and the other three times I’ll do over the telephone. We can talk about the same stuff. I can order the same stuff. If you have a concern that you want me to examine, obviously I’ll need to see you or look at it on video or actually see you. But most of this stuff we can do.” And I think the most powerful message comes from their doctor who they trust, but we are going to have to sell that to them probably face to face at first. And that’s what we’re advocating for.* –Physician *We’re actually saying, when you start talking to your patient about their patient experience that’s coming up in their initial evaluation, your messaging really needs to be “We will be doing almost half of the visits by video and we need to do this in order to be able to keep the environment safe for you when you come for your in-person visits.” So we are going to have a very different approach to how we structure our in-person visits vs. the video visits. The in-person visits really need to be deliberate and intentional*. -Physical Therapist *There’s a lot of people who said, “I would [do video visit] if I had to, but I’d still prefer in-person.” And that’s the group of people that we’re going to have to circle back to and say, notwithstanding that, we’re going to be doing it by video.* -Physical Therapist *…even though we think clinically it’s okay to do a video visit, we realize that on the patient side, there may be some hesitancy. And also a matter of perspective of whether they’re getting the same quality of care. We’ve also been learning that they actually do like video visits much better than phone visits for those that can do it, which has been good.* –Physician *I think it’s going to be a culture shift for our patients. It’s been a culture shift for the nursing staff and providers. It’s going to be a culture shift. Many of them want to be seen face-to-face …So it’s getting them comfortable in realizing it’s the same level of care*. -Nurse *There’s so much more work around educating patients and staff. And really not just educating patients and staff, but making sure that patients desire, feel comfortable with, and can benefit and engage in a video visit. And have the technology and tech literacy, as well as the health literacy to do so that we think warrants a lot more work upfront maybe.* –Physician *And some of the older folks, too, they’re very traditional, they’re very leery about technology. They don’t want their “information” out there, so they don’t want anything to do with our video visits. They’re afraid it’s going to go viral somehow, so they absolutely do not want video visits. So that’s a challenge, educating them that it’s a secure link, that their information won’t go anywhere, so that’s another bit of education.* -Nurse

CLINIC/PROVIDER Inner Setting	Staff telemedicine training/empowerment	*… we quickly discovered that this was a new technology. People had not tried it yet. And they really needed to have mentoring experiences at least for the first one and if not for more*. -Physical Therapist *… we still have staff members a month later that have not yet done a video visit. And it’s partly because of their own fear and fear of new and fear of technology. And really just it’s not something that we learned in school. Is two hours of training really enough? It really isn’t. It’s really about learning a language.* -Physical Therapist *The second bucket is we have a digital divide within generational workforces. So not everyone who’s working in our integrated health systems in terms of our employees—from nurses to providers to clerks—not everyone’s at the same digital-savviness level. So not everyone can learn texting or understand Zoom in the same manner. So they also need an update.* –Physician *I think one of the biggest barriers is going to be staff readiness and comfort with it. Because even if patients are excited and want to do it, if the staff aren’t comfortable, it’s not going to go well; and if we don’t train them well enough, if we don’t equip them with the right personal skills as well as technology to do these well, they’re going to get frustrated with it and not see the value in it and it can flop. If the staff don’t want to do it or don’t feel comfortable doing it, then it’s not going to work. So I think that’s one of the areas we need to focus a lot on is proving the value, giving them the skills and the training, and giving them guidance on how to select which visits are best and then how to optimize those visits when you’re doing them, and how to do them efficiently so that you don’t feel like, oh, I did a phone visit with a patient, but I still need to see them in person. That defeats the purpose of what the video visits are supposed to do.* –Physician *Because it was a small group, I was assigned do the actual training and implementation. So I actually went out and trained the nurses on how to access and how to actually use the Zoom because there’s some technology that they have to maneuver through.* -Nurse *We didn’t ask that it [champions] be an RN, an LVN. It could have been a CMA. In the original area it happened to be my LVNs that stepped forward and I think they just wanted to be that subject matter expert. I think for them it had an internal award, not an external reward. And to get to go to another clinic and share their knowledge, I think it was just their personality.* –Nurse *Remember like each clinic, we base it—based on their attitude and the willingness—number one, willingness to participate. They’re, of course, technology savvy. So that’s how we—each clinic we already know who they are. What we do is just after the implementation, those nurses that participate then go to the next clinic just to support them and to train them in the beginning. It’s like train the trainer, you know?* –Nurse *Because they’re very savvy, technology savvy, that’s one. And second, great attitude and team player attitude and just, yeah. We know who they are.* –Nurse *It’s like certain staff that you know are always pioneers, always trying to champion through just other things that they were asked do you want to be part of this, and they say yes. So it’s more like the people that have the good rapport already, have already championed in different aspects. And we just select them and they carried it on very well. They are very strong.* -Medical Assistant *… I made available to anyone who was interested and our telehealth accelerator team basically laid it out very clearly saying, “This is an imperfect science. Okay, there’s not a standard, if you want to do it, we’re going to expect you to develop the standard, but you have to work through all the challenges.’ So we had a set of individuals who took on that role and they were magnificent. They were unbelievable. I mean, I was there for them, obviously, but I consider them the heroes of the pandemic. So they’re very good.* -Physician

	Standardized clinic workflows to facilitate telemedicine uptake	*But in terms of dermatology, podiatry, ophthalmology, I don’t exactly know how they’re deciding which patients are—which disease states are good for Zoom and which ones would need to come in person. I think in pediatrics it’s just anybody who doesn’t need to come in person like for a physical exam. And a lot of times in primary care basically we’ve just been using the telehealth visit as a way to filter out face-to-face, so we don’t make that face-to-face decision until the telehealth visit has been done. But in peds, that’s basically been the model, unless we know that you need certain vaccines or a newborn exam or a well-child physical exam for back to school, et cetera.* –Physician *So the determination of whether a visit is appropriate for virtual, phone, or video vs. face-to-face is made by the provider, which is either a resident trainee or a faculty attending.* –Physician *We had to ensure that the providers would scrub the list of patients prior, to see which ones were actually appropriate for a video visit as opposed to a face-to-face visit. Because obviously, depending on what the condition or diagnosis was and what information needed to be—whether or not a face-to-face was more appropriate or a video was appropriate.* –Nurse *Yeah, that process [patient selection for telemedicine] is more like the provider and leadership. They made an outline of what’s a good criteria to select these patients. I’m not part of that—yeah, we just get the list. But it is through leadership with providers. They do have a criteria that makes them qualify for it, because not everyone is qualified to do the Zoom, apparently, yeah.* -Medical Assistant *Well, that’s when we can send them the link with the information on how to download the Zoom. In the beginning though, the nurses, like I said, we were just learning; the nurses were doing it themselves. Walking the patient through. And then, as I said, it was all about patient selection. If patient said “Well, I’m not comfortable with that,” then we moved on and found a patient who said, “Oh yeah, I already do Zoom because of school or my child does, I know all about it.” In those patients, it was easier to work with.* –Nurse *But what we are doing is the providers in that particular specialty clinic are identifying patients who they feel are appropriate for a video visit. When we first started, what the nursing staff had to do was actually reach out to the patients that the providers identified. And call the patients and ask them if they would be willing to do a video visit as opposed to either a phone visit or a face-to-face. And we tried to explain to the patient the benefit of the video visit. That it would be easier on them because they wouldn’t have to drive all the way over here. They wouldn’t have to find parking. You know, just selling all of the positive points. As patients agreed and consented to video visits, then the nursing staff had to go in and actually change the appointment time to a video visit appointment time.* –Nurse *And what we’re actually going to try doing too is—and we’re not sure about how this will work, but this is something we’re looking to see is screening for level of appropriateness. It’s one thing to think clinically that, yes, a video visit is fine. But the other part is on the patient side—if they don’t have someone that help them manage a smartphone or manage a video visit, it’s not going to be successful and it’s only going to frustrate the patient and the staff. So we don’t want to set that up. So we’re actually trying to figure out like is there a screening tool that we could use.* -Medical Assistant *And what I’m hearing from the staff in terms of the workflow, that had to be part of our clinical workflow, that we don’t know is do they have Wi-Fi? Do they have a cellphone? Do they have a computer? So we don’t know that. We don’t know that kind of demographic. It becomes a responsibility of the clinic. And how do you know? And even if you had all that, like they miss their visit. They don’t know. Their battery ran out. How do you do that? So that part of the workflow is two or three boxes on a swim lane. That should be a massive (challenge? @ 00:27:42). So that’s missing. So to me that’s a whole different category. And who are the owners of that? Who would be the best to really assess that? We don’t have a workflow for that. So I think with a better understanding and segmentation of those type of patients, it would make our workflow a lot easier.* –Physician *And I believe that all the training material was really done nicely, but it was done by a team of nurses—very good nurses, by the way—and great material, but it was material that was not developed by maybe getting some end users, some nurses that are on the line to bring those kind of questions. Is there an assessment criteria that we can kind of screen our patients with to see who are the candidates, rather than just kind of doing a cold call. So that I believe lacked, but not minimizing that the development of the training material and all the job aids and things that—I want to say the DHS nurse informatics from ORCHID did were wonderful, wonderful, great material. When we do really go back to—and we’re getting close to going back to the drawing board, bringing all those materials in, but also adding, you know what? We need not a cold-call list. We need to be able to really identify when we go to that end user and say, “Hey, let’s talk first before I tell you you're going to implement something. How would you stratify your patients that could qualify or be willing to do a video visit with you?” And that way they don’t feel so unsuccessful.* –Nurse *… we’re actually trying to see if there's a more objective way to do an assessment in advance so we know the patient’s level of readiness in advance. There are some that are out there and there are some that other sites around the state that are part of the grant have used. I don’t know that any of them are validated or anything like that. And some are I think too long to really be useful. So my goal out of this would be for us across DHS that we can come up with a three- or five-question screener that even a nonclinical person like a call-center staff could provide so that if someone calls and says, “I want to do a video visit,” the scheduler, who’s separate from the clinic, could say, “Okay, do you have a cellphone or a device that has internet? Do you have Wi-Fi in-home or do you have somewhere where you can go to do it? Are you comfortable doing it on your own or will you need some advance help to make sure you’re ready?” Something very simple like that and it wouldn’t keep someone from being able to schedule a video visit as long as they have at least the phone and Wi-Fi, but it would at least give the team a heads-up as to what the person’s level of comfort is with doing the video visit so they know in advance whether it’ll be a quick logon and just go or whether they may need some extra time to make sure the person’s ready or maybe they need to do a practice session in advance of the scheduled visit, something like that.* –Physician *So I’m working on developing criteria of let’s say you identify a patient that you're scrubbing a chart and clinically it makes sense to do this as a video visit because there's something you want to see visually. And once you have identified that patient, rather than saying, “Oh, this is a good candidate for a video visit or not and deciding accordingly,” saying, “This is a good candidate for video visit versus this is a candidate for a video visit that is going to probably need some help and let’s make sure we provide whatever help and resources are needed,” or be prepared that they're going to need help* -Physician

HEALTH SYSTEM Process	Technology planning	*It’s like lighting has to be good, the quality of the video, so that the appointment or whatever the doctor needs to do as far as visually seeing it, can maybe get a better plan for care.* -Medical Assistant *As long as you have the equipment and enough equipment to make these video visits happen, then that’ll be more beneficial. And then it’s like scheduling because then if you have the equipment, then you can schedule more patients to do it. But if you have one or if you have none, or if you just have a tablet, it’s harder too. Luckily we were able to get these WOWs (workstation on wheels), which are very beneficial because it has a better screen, it has better video quality, and its sole purpose for this whole computer is just for video visits itself. And it’s easier for the staff to just recognize, oh, this is for the video visit. And just this morning, I’m using one but the staff already knew where to go. They just knew that you just pick it up from this designated area.* -Medical Assistant *Like I said before, no matter how much we’ve been doing it, or even the superusers for each clinic, there’s always an issue with sound or it’s something about “I can’t hear you,” or “Can you hear me and is the volume of the screen up?” Because we actually came across that where many times the reminder call from the staff nurse would call the patient and they said, “Everything’s fine, okay, we’ll see you tomorrow.” And then come the day of, something happens with the volume. And it’s just a bunch of troubleshooting like that*. -Medical Assistant *Facility-wise it’s just as far as if they have the equipment to do it, which I hope isn’t an issue if they do want to spread it clinical-wise. And then the manpower for the teams to support, to teach these things at every—because whenever we do a go-live, we all have to be there—and let’s say we’re at our fifth go-live but yet there’s still four of the other clinics that are still continuously doing video visits, it’s like, well, we need to focus on the go-live, but then we also want to help with these four other clinics. How do you spread the manpower to help with all this stuff? So that might be a barrier as far as staffing goes on that. And then teams, multiple teams will be involved, so IT and stuff, so their manpower, nursing manpower, leadership manpower.* -Medical Assistant *And we are still struggling with the best way to check in patients and have the CMA and the ancillary staff involved in the visit. Because some of our providers, actually two days out of the week our providers are actually at their houses doing telephone visits. And eventually we’ll have to figure out, okay, are we going to do video visits while they’re at their house? But when they’re not on site, my staff, their ancillary staff, is actually on site. So the handoff between the two is still challenging. And I know some of the clinics are a little bit more I would say maybe ahead of us as far as involving the CMAs and the LVNs and they’re doing the same intakes that they were doing face to face. And I don’t know what the best way is to do that yet.* –Physician *So setting up space or the reengineering of space has been rather involved, because these spaces were not necessarily readily available. So we’ve really had a domino effect of really reengineering the use of space and also a lot of clinical moves themselves to best utilize the space available within our hospital building. So stakeholders who I—thankfully, I think we had the necessary support to move this forward and as element come along or as needs come along, ad hoc members such as facilities and IT are brought in.* –Physician *One of the challenges I think, though, is because we still are having a combination of face-to-face, phone visits, and the video visits, it’s again a provider thing where they need to figure out, well, who’s doing telemed, who’s doing the face-to-face, who’s doing the phone. It’s that on their end where it might be a little difficult because you still have your patients that are here face-to-face and they need to be taken care of just as well. So it’s the juggling act of finding that happy medium of how to assign out and how to get all of that taken care. It’s, to me, a little difficult, just like our nursing assignment. We have to figure out who we’re assigning for phone visits and the face-to-face that we still have, and that there’s visits and all of that. It’s just a new normal that we’re all trying to figure out right now*. -Nurse *One thing we’ve been doing here is trying to create nonclinical spaces to do telehealth, so like telehealth hubs. And so that I think is a big piece of it is you want people to be on campus and not be remote at their house. You have to build these computer labs or telehealth labs so that the expensive clinical spaces that are designed for clinic use don’t have to be used for telehealth. Or, alternatively, you do make remote work more possible and equip at least providers with the equipment to be able to do this from home. And then have a support structure on the nursing side. That would probably still require telehealth hubs for nursing to support the providers who are remote. And then I think the other piece of it is probably the software. I don’t think Zoom for Health has been scalable software for DHS because it’s a lot of management of the licenses and the accounts. And so having a separate system is always added work. So not only do you have to schedule an appointment in the EMR, but you have to schedule a license and you have to link the two.* –Physician *So what are the standard elements to creating a telehealth workstation, whether it be having a dual monitor, a singular monitor, having headsets, landline versus, I guess, voice, or having Wi-Fi versus wired connection, all of those elements to kind of understanding what is the best set up for telehealth so that it can be replicated in these kind of shared remote locations.* –Physician *Well, we're going to need to do video visits, let’s place some orders,” everything was already backordered. We had to get DHS as a system just to meet the needs of our system. We already had a big delay in getting the hardware available. I want to say when we were looking at placing the order early on in the pandemic, it was probably a two-month, maybe three-month backorder. And so that took some time to even just get the hardware in. So even if you had licensing to do a visit, you didn’t have the camera*. -Medical Assistant *There are also challenges with connectivity. The Wi-Fi is not optimal. Even when we have our own internal meetings on Zoom or on Teams … we'll have times when we're on some meeting and the person suddenly is talking all garbles. And you know they're talking, but you can't understand a word. So there's just all those types of things. And I think at DHS, at least at Harbor the Wi-Fi isn’t really up to the level that it needs to be to be able meet the challenges.* -Physician *And so it took that long for us to be able to interface effectively with the medical records system. They had to do a completely new build for us for the billing part of it, but it is now done. There's a lot of stuff on the scheduling side that’s still not working properly, but it’s at least not preventing us from billing.* -Physical Therapist *So we wanted to make sure that if we put forward a video-visit model, that it would be compliant with HIPAA and whatnot going forward. So I think that’s why we went with the license model that we have and that obviously when you go with that kind of license model, albeit, that kind of said we had to prioritize who needed to go first … and also to train everybody to do this workflow. So that right there probably set the tone like where things had to go*. -Medical Assistant *And so having a separate system is always added work. So not only do you have to schedule an appointment in the EMR, but you have to schedule a license and you have to link the two. And so I think this is where DHS is moving is to more of an integrated solution for the EMR so it’s more simple and you can just click on the patient chart and open a video platform and, on the patient side, they’ve downloaded whatever they need to download. I think that would be a little bit more scalable* -Physician *I think probably bringing in some of the end users to develop some of the—or just have an open conversation with some of the end users to really see what do they think. Especially bringing in the ones that we implemented already; not bringing in people that don’t know, but people that—like the three sites that I’m—especially the two sites—maybe interviewing those care managers, bringing them in on a session and saying, “Hey, look it, we have this plan. What do you think we could do?” The end user is always so vital in this conversation for success. We can always make the decision that we’re going live, but if we're not there on the ground, you know*. -Nurse

	Leadership communication and goal setting	*Secondly is I think it’s very important to have a bidirectional understanding of who’s really going to be your implementation teams and give them channels to give you really quick feedback on things that practically can and cannot happen as you implement. I think we’re in a pattern of actually just rolling things out and specifically I think in probably the initial rollouts, we already have very clear signs of what is probably going to be problematic and not and we’re not agile enough to process that information to really change either our approach or maybe potentially our overall strategy, but instead we will just focus on the outcome.* -Physician *I think the big barrier is just that if there’s no leadership buy in or it’s not organized, then the people will not buy in. That’s one. We have to make sure that every key stakeholder are right there from the beginning and the expectations from each and every one of them, clear expectations and then support. What do I mean by “support?” Being there, being present, communicating, providing them with the tools if they need it. One of the support is just having educators a lot. It’s a big support, knowing that they’re there, that they’re not alone. The bottom line is we explain the why, and people understand that this is for—this enhancement and also good for our patients. So we do a lot of explanation. We communicate a lot at Harbor as far as my team is concerned, a lot of checking in with them. That’s very important to me. To me, any project that we implement at Harbor, we do the same thing.* -Nurse *The other related issue is that strategically the way that the DHS highest levels of leadership have really pushed to see the implementation rolled out I think has been—I understand why they made the decisions they made, but from a practical implementation perspective, it doesn’t make any sense.* -Physician *And I see that as a resource-utilization mistake because if the goal is to get this high-value clinical resource to as many patients encounters as possible, giving it to the people who are really excited about using it and are going to make it work will get it to the most patient encounters as possible. Giving it to whatever specialty you deem through some esoteric process was high priority, yeah, sure, if there are providers in that specialty that are really excited about, go for it.* -Physician *So when you say “telehealth,” are we trying to get to standard ubiquitous implementation? Are we trying to address use case on our needs? And those have to be well thought out from the beginning so that people know how to resource and design the appropriate outcomes and the KPIs to keep all the downstream teams understood. So communication I think is key. So that’s number one, I think just having the appropriate mission and scoping it appropriately.* -Physician *So one of the things that I would say—and hopefully this doesn’t go too far throughout anywhere else, but often we come up with a solution before we’ve really found the problem.* -Physician *And then the other thing for success is being very clear, what is the goal? What are we trying to achieve? And who’s going to benefit the most? Are we just looking at numbers or are we really looking at what is our goal to reach this patient and see improvement in their relationship with us and trusting us? And one day when we go back to real clinic time again, keeping them engaged and keeping them healthy. Yeah, I think that’s a lot that we need to look at first*. -Nurse *So we need to understand before the in-person visit even happens what it is specifically that we are trying to accomplish in person that we could not accomplish by video, because those things should happen by video.* -Physical Therapist

### Patient level themes (CFIR Outer Setting)

Patient level themes corresponded to the CFIR “Outer Setting” construct, detailing conversations regarding patient needs and resources and patient influences in implementation feasibility.

### Patient preparedness

**Figure 1:**
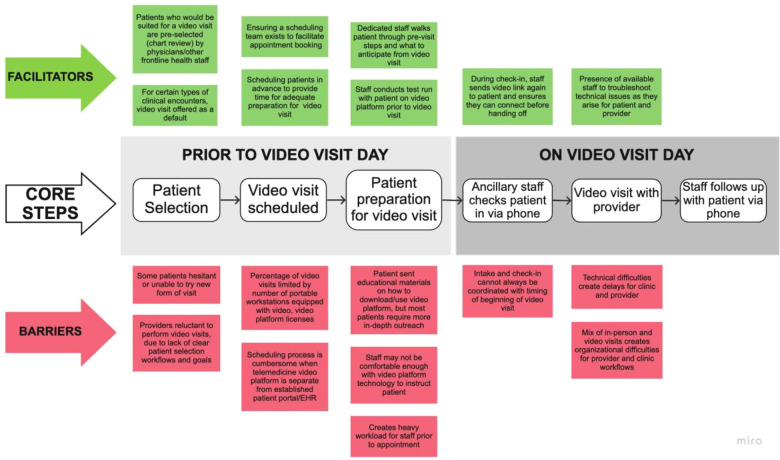
Process map for typical LAC DHS clinic video visits.

Conversations about *patient preparedness* encompassed digital health barriers in the patient's context that could affect the feasibility of telehealth for the safety net. Stakeholders described barriers to use of existing digital health tools due to a “digital divide” for their populations, characterized by low digital literacy, limited broadband and/or cell phone data plans, and lack of access to Internet-connected personal devices (and/or high quality digital devices). Other technology-specific challenges included patient usability of the LAC DHS telehealth interface and the English-only language of the current platform. Stakeholders noted that the current video visit system required multiple steps on behalf of the patient (see Figure 1 process map), which were challenging for most patients. Furthermore, a “successful” (i.e., completed) video visit usually depended on many staff phone calls in advance of the encounter. This was noted as not sustainable for staff given constrained resources and time. Some clinic staff indicated that their clinics had resorted to “selecting” for patients who they thought could complete a video visit, and promoted it among these groups. However, they also noted that this was not equitable, as it was unfair to promote video visits among a subset of patients—when potentially all safety net patients could benefit from the service.

### Patient acceptability

Stakeholders also discussed *patient acceptability,* with specific conversations centering around a culture shift with regards to telehealth. Stakeholders shared how so much has been taken away from the large proportions of racial and ethnic minority communities served by the Los Angeles safety net—due not only to COVID-19, but also because of historical and contemporary systemic injustices. The threat of losing a personal connection to their doctor and health system was a perceived fear from their patients. Stakeholders suggested eliciting and directly acknowledging concerns about telehealth with patients, and then following up with discussions about benefits as a way to assuage misconceptions while shifting the patient culture about expectations for clinical visits. The goal would be to normalize telehealth visit offerings as part of high quality clinical practice.

### Clinic/provider level themes (CFIR Inner Setting)

Clinic and provider level themes corresponded to the CFIR's “Inner Setting” construct—detailing issues regarding clinic characteristics such as networks and communications, workflows, culture and climate.

#### Staff telemedicine training and empowerment

Participants felt that leadership assumed that frontline staff, who interface the most with patients, would be familiar with telehealth and able to promote it. However, this was not always the case, as some staff were not comfortable with the technology and had not used telehealth visits or a patient portal themselves. Participants discussed the need for more recognition of the different comfort and electronic health literacy levels of staff and medical providers and endorsed a need for effective training during implementation (including “refreshers” or updates about the technology) and immediate contacts for troubleshooting problems. In these discussions, participants talked about identifying ideal staff to serve as telehealth “champions,” delineated characteristics of champions, and ways to support/reward champions, that would help in telehealth promotion and facilitation within clinics.

#### Standardized clinic workflows to facilitate telehealth uptake

Stakeholders discussed the informal use of workflows to identify appropriate clinical scenarios for telehealth. In the video visit pilots, this was being done *ad hoc* by physicians and other medical staff. It was stated that systematic identification of clinical visit types ideal for telehealth (medicine reconciliation, diabetes coaching, treatment discussions) could potentially be created, with algorithms or workflows to automatically match these to telehealth offerings (especially within each clinical specialty). Participants mentioned that such efforts could save time for nurses and doctors. Staff thought that automatized offerings could promote telehealth uptake, as patients would probably be less proactive about asking for video visits, given their low baseline awarenes and knowledge about telehealth in the safety net. The other major operational workflow that participants mentioned was the need to integrate digital readiness patient screening or assessment tools as part of clinical care. During early phases of implementation, providers spent time trying to “identify” ideal candidates to offer a telehealth visit to (e.g., those registered to a patient portal, had an email account on file in the health record, or who used apps on their phone). While this identification process was somewhat helpful to increase the yield on completed telehealth video visits, obtaining this information required extra time during the visit and limited the service to subgroups of patients. Participants brought up the potential of digital screening tools to increase immediate yield of visits and help identify patient needs and allocate resources for support. For example, a pre-visit preparation session could help make video visits more accessible to those who most needed it and troubleshoot connectivity and other problems. However, participants noted that this screening process would require a standardized tool, such as a set of validated predictor questions that could quickly and easily identify patient's needs for support. Without a uniform approach and systematic guidance, too high a burden would be placed on staff and providers.

### Health system level themes (CFIR Process)

Themes that corresponded to the health system mostly reflected CFIR factors regarding the planning, engaging, execution, reflection, and evaluation over telehealth implementation.

#### Technology planning

Among the barriers cited were lack of planning for needed equipment and ancillary resources: such as not enough computers with cameras in the clinic, lack of physical space to expand telehealth given constraints of a simultaneous in-person and telehealth visit schedule for providers in a county clinic with a shortage of clinic rooms, need for differentiating telehealth visit procedures from in-person visits (e.g., a required “nurse intake” for a telehealth visit may not be useful), challenges with broadband Internet access in clinic such as poor Internet reception in some of the county clinics, lack of systemwide IT support to facilitate early implementation, and video visits not integrated with the system's current patient portal.

#### Leadership communication and goal setting

Participants endorsed the importance of routine telehealth discussions between leadership, IT, and front-line staff, especially for the sharing of best operational practices, digital educational tools for patients (and staff), and patient-centered digital engagement across different sites in this large county health system. Most participants recommended that the health system invest more to support patient education that impacted preparedness for telehealth access and high quality utilization. They described feeling like they were designing these processes in real time. Additionally, although tailoring to their specific settings is needed, they would have benefited from learning about other clinical sites' models, approaches, and “mistakes,” particularly those sites that started earlier with telehealth implementation. They called for LAC DHS to develop more centralized patient and staff telehealth educational resources.

Participants also discussed being “unclear” about the goals of the implementation, expectations from leadership and what telehealth “success” looked like in the safety net. Did this mean that all patients should be using video visits or a goal number or percentage of visits by some date? Would these metrics be uniform across specialties and clinic sites? The stakeholders we interviewed felt it would be important to have staff partner with leadership in asking these questions and in goal setting. They also emphasized the importance of transparency about specific telehealth goals and regular updates to their frontline staff. Participants also mentioned that these metrics should be set with the overarching aim of offering the highest quality of care possible to their patients and not for the sake of simply reaching some arbitrary telehealth uptake or usage metric at LAC DHS. Finally, participants noted that without measurable telehealth goals, objectives and metrics, there was less incentive and accountability to continue the use of video visits beyond the pilot phase.

## Discussion

This qualitative study of informed safety net stakeholders presents some important multilevel lessons learned around telehealth implementation in settings caring for historically and contemporarily underserved patient populations. Of note, thematic narratives from these interviews highlighted the following takeaways: patients' longstanding risk for and history of the digital divide must be addressed up front, with the aid of trained multidisciplinary teams, established workflows tailored to each clinical setting that facilitate telehealth, and with clear communication and partnership between safety net leadership and on-the-ground staff about the objectives and goals for telehealth care for their patients. Most participants stressed the need for the health system to invest more resources to support patient engagement and education that impacted preparedness for telehealth access and high quality utilization. If these challenges are not addressed, this could be a set-up for worsening health disparities for these patients (not only LA safety net patients, but all over the US), already at higher risk of poor disease management outcomes.

Our study does reinforce the findings of prior digital health literature regarding the need to better understand patient preparedness for telemedicine. This includes several dimensions for digital access and uptake which have been described in the digital divide literature addressing safety net patient populations—(51–67) including devices and data plans as well as clear expectations for digital skills from more basic (opening an app) to more sophisticated skills like finding, sending, and/or interpreting health information digitally (68–73). Discussions of digital health equity must also address patient acceptability of telemedicine, with specific conversations centering around a culture shift for patients. The safety net serves a large proportion of minority patients, with populations that value a personal relationship with the doctor (i.e., “*familismo*” in the Latina/o/x community, a primary ethnic minority population served by LAC DHS) (74). The introduction of video or phone visits is an adjustment for patients, and requires reassurance that their patient-doctor relationships would be conserved, something that has been noted in early patient portal studies within the safety net, especially among Black patients (75).

Our study adds new content around the readiness and culture of providers and staff to deliver telemedicine, which is not actively explored in previous literature. Participants discussed that there was almost an assumption made by leadership that frontline safety net staff interfacing the most with patients would be comfortable using these new telehealth tools. Future implementation will have to focus on the technology needs and understanding of the workforce, and staff telehealth training and empowerment for those at the front lines of the health technology implementation in the safety net (76). Also, much of the discussion at the clinic and provider level revolved around the extra use of resources to identify high quality clinical scenarios and appropriately identifying what support patients needed to use the video visit service, to meet these unmet digital needs (52, 77–79). In a busy and under-resourced setting, the concept of standardized clinic workflows to facilitate telehealth uptake is an approach for this, and has been recommended by other organizations who have focused on digital health uptake in safety net settings (79).

Health system-specific challenges in the discussions encompassed technology planning barriers like usability and the English-only language of the video visit platform, which has also been previously discussed in the literature as major factors (80–83). Participants mentioned the lack of infrastructure in clinic spaces and the incompatibilities with concurrent in-person and telehealth visits functioning efficiently for a provider in the same clinic workrooms and nurse teams. In terms of leadership communication and goal setting, there was a clear need for more partnered goal setting around telehealth implementation, and transparency of process. Of note, these early stakeholder discussions have led to the development of the LAC DHS Virtual Care Workgroup, a consortium of LAC DHS primary care clinic directors, patients and community advocates from Patient Family Advisory Councils (PFAC's), interpreter services, social work and chronic disease health educators. This workgroup has been meeting every week since the spring of 2021 to help shape LAC DHS local telehealth policy and determine the evidence-based interventions needed to appropriately deliver effective telehealth. Some recent innovations borne out of this workgroup include: addition of telehealth (video visits, patient portal) tutorials into educational content delivered by health educators in their chronic disease self-management classes for LAC DHS patients, and the development of a health technology navigator community health worker corps with health technology navigators “stationed” at various LAC DHS clinic waiting rooms—helping patients enroll and use their portal and become familiar with other telehealth offerings while they wait for their in-person appointments.

Study limitations included the small sample size of staff and leadership who were nominated to participate in the interviews, and limited generalizability to safety net health systems outside of Los Angeles County. This study focused on workforce and leadership stakeholder perspectives in the implementation—however, research centered around patient perspectives will be needed to validate changes, and advance substantial progress. In addition, this study did not address the relevant role of external policies like Medicaid reimbursement of phone and video visits, impact of patient-centered staff such as health educators and community health workers (who have been shown to improve digital health uptake in these settings), and availability of telehealth platforms and technology support in multiple non-English languages—all of which have been shown to be critical factors in the sustainability of telehealth in the safety net (84). Future research will need to more fully explore these in safety net settings to ensure that evidence-based factors that affect long-term high quality telehealth for these patients are addressed.

Overall, the purpose of this formative study was to generate initial health stakeholder insights regarding meaningful telehealth implementation for safety net patients and settings, as all health systems around the country are integrating remote strategies to reach out to patients, even beyond COVID-19. Although telehealth is not a full replacement for all in-person health care, it is important to note that telehealth is an added and complimentary clinical tool to address longstanding and continued health access inequities among underserved patients, if implemented appropriately in these settings. Because Los Angeles County is the largest and most ethnically diverse county in the country, these unique insights will be applicable to safety net systems across the US who are working to augment access to care and prevent worsening of chronic disease conditions among their patients. This augmented access will be especially needed after the disproportionate physical and mental health traumas inflicted upon this safety net population from COVID-19—effects that will resonate years beyond the end of the pandemic.

## Data Availability

The raw data supporting the conclusions of this article will be made available by the authors, without undue reservation.
